# Elevated Blood Lead Levels in a Pregnant Woman and her Family from Traditional *Kansa* (Bronze) and *Pital* (Brass) Metalware — New York City, 2024

**DOI:** 10.15585/mmwr.mm7418a1

**Published:** 2025-05-22

**Authors:** Paromita Hore, Kolapo Alex-Oni, Slavenka Sedlar, Nevila Bardhi, Jacqueline Ehrlich

**Affiliations:** 1 Bureau of Environmental Disease and Injury Prevention, New York City Department of Health and Mental Hygiene, New York, New York.

**Summary**

**What is already known about this topic?**

Traditional metalware can contain high levels of lead, which can leach into food and drinks.

**What is added by this report?**

In July 2024, blood lead screening in New York City identified a pregnant woman and two family members with blood lead levels above the Council of State and Territorial Epidemiologists’ reference value of 3.5 *µ*g/dL. Elevated lead levels were found in traditional *kansa* (bronze) and *pital* (brass) metalware from Nepal that the family used to prepare and serve food and drinks.

**What are the implications for public health practice?**

Clinicians and public health professionals should be aware that imported metalware from around the world can be communal sources of lead exposure when used to prepare or serve food and drinks.

## Abstract

Lead exposure, even at low levels, can cause detrimental health effects across all ages. The New York City (NYC) Department of Health and Mental Hygiene receives blood lead results for NYC residents who are tested for lead and routinely conducts investigations to determine sources of lead exposure. In July 2024, blood lead surveillance activities in NYC revealed high levels of lead in traditional *kansa* (bronze) and *pital* (brass) metalware from Nepal. Use of these metalware items for preparing and serving food and drinks was associated with blood lead levels above the Council of State and Territorial Epidemiologists’ blood lead reference value of 3.5 *μ*g/dL in a pregnant woman, her spouse, and their child (range = 6–18.7 *μ*g/dL). Clinicians and public health professionals should be aware that traditional metalware from around the world can contain high levels of lead, and when used to prepare or serve food and drinks, can be communal sources of lead exposure.

## Introduction

Persons of all ages can experience detrimental health effects of lead exposure, even at low levels.* In New York City (NYC), lead exposure is commonly associated with lead-based paint, especially among young children who engage in hand-to-mouth behavior, and with occupational lead hazards among adults who work in the construction industry. Through routine surveillance activities, the NYC Department of Health and Mental Hygiene (DOHMH) has also identified various consumer products from around the world containing high levels of lead, including traditional ceramic and metalware used by members of many communities for preparing or serving food (*1*).

## Methods

New York law requires that health care providers conduct blood lead testing for pregnant women determined to be at risk for lead exposure, based on the recommended assessment at their first prenatal visit.^†^ Blood lead testing is also mandatory for children aged 1 and 2 years and adults with occupational lead exposure.^§^^,^^¶^ The NYC DOHMH receives all blood lead results for NYC residents who are tested for lead (*2*). Follow-up actions are initiated at threshold blood lead levels (BLLs) of 3.5 *μ*g/dL for children and pregnant women and 5 *μ*g/dL for nonpregnant adults.** NYC DOHMH also recommends testing household members of persons with BLLs above the threshold. During follow-up investigations, NYC DOHMH administers a risk assessment questionnaire to identify potential lead sources and, when applicable, conducts environmental testing for lead in paint, dust, and consumer products such as spices, cultural powders, and health remedies (*2*). Measurements of lead in paint are conducted using a Viken Detection handheld x-ray fluorescence (XRF) device and dust wipe samples are analyzed for lead by an accredited laboratory using Environmental Protection Agency method 7000B.^††^ Samples of consumer products suspected to contain lead, and which might be mouthed or ingested, are collected and sent to an accredited laboratory for analysis using the appropriate analytical methods. Certain consumer products such as ceramic or metalware are also screened for lead using the handheld XRF device before laboratory testing. Lead test results, risk assessments, and case notes are stored using a proprietary structured query language server database (*3*).

In this investigation, 33 XRF paint measurements and six dust wipe samples were collected for lead testing. In addition, 17 consumer product samples were tested for lead: spices (six samples), powders used for religious purposes (two), and imported metalware used to prepare or serve food and drinks (nine). The spices and powders were analyzed for lead using Environmental Protection Agency method SW6020.^§§^ Metal cookware and dishware, which included four metalware items, were screened for lead using the Viken Detection handheld XRF device, and when feasible, analyzed for leachable lead using the ASTM International (formerly the American Society for Testing and Materials) C738 standard test method^¶¶^ per Food and Drug Administration (FDA) guidance (*4*). Lead results were compared with regulatory standards or available reference limits. The regulatory guideline for lead in paint is 0.5 mg/cm^2^, and the guidelines for lead in dust are 10 *μ*g/ft^2^ for floors, 50 *μ*g/ft^2^ for windowsills, and 100 *μ*g/ft^2^ for window troughs. The reference limits used as guidance for lead in the spices and powders were 2 ppm and 10 ppm, respectively (*5**,**6*). The metalware leachate lead concentrations were compared with the FDA regulatory guidance for silver-plated hollowware, which is 7 mg/L (*4*). The NYC DOHMH institutional review board determined that this activity did not constitute human subjects research.

## Results

In July 2024, prenatal testing detected a venous BLL of 11.2 *μ*g/dL in an asymptomatic woman aged 28 years, who was 11 weeks pregnant. Venous BLLs were 18.7 *μ*g/dL for the woman’s asymptomatic spouse, aged 27 years, and 6 *μ*g/dL for their child, aged 7 years. Risk assessment interviews revealed that the pregnant woman’s spouse had lived in the United States for 5 years. The spouse traveled to Nepal in April 2024, at which time the pregnant woman and the child returned with him to the United States.

None of 33 XRF paint measurements or six dust wipe measurements collected at the family’s home exceeded the regulatory guidelines for paint or dust. No lead-based paint hazards were identified in the child’s school. Lead concentrations in the spices and powders used for religious purposes were below reference limits. However, four of the nine metalware items referred to as *kansa* (composed mainly of copper and tin [bronze]) and *pital* (composed mainly of copper and zinc [brass]) (*7*), that the family had purchased in Nepal, had XRF lead concentrations exceeding NYC DOHMH’s reference limit of 0.4 mg/cm^2^; the leachate lead concentration for three of these items far exceeded FDA guidance limits for similar products (e.g., silver-plated hollowware) (Figure). The four items included a large cauldron (XRF lead concentration = 5.2–15.1 mg/cm^2^ and leachate lead concentration = 18.6 mg/L); a small cauldron (XRF lead concentration = 4.7–9.9 mg/cm^2^ and leachate lead concentration = 41.4 mg/L); cup 1 (XRF lead concentration = 13.7 mg/cm^2^ and leachate lead concentration = 309 mg/L); and cup 2 (XRF lead concentration = 3.7 mg/cm^2^; leachate lead concentration was not tested). 

**FIGURE:**
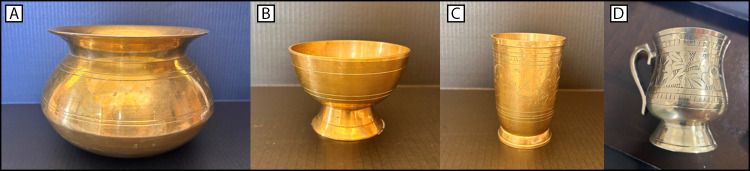
*Kansa* (bronze) and *pital* (brass) metalware* used by three family members with elevated blood lead levels — New York City, 2024 Photos/New York City Department of Health and Mental Hygiene * (A) Large cauldron used for preparing foods or liquids, (B) small cauldron used for serving foods or liquids, (C) cup 1 used for serving water during meals, and (D) cup 2 used for serving tea. All metalware was purchased in Nepal.

NYC DOHMH advised the family to stop using the implicated metalware products. By September 2024, BLLs decreased to 1.7 *μ*g/dL for the woman and 10.7 *μ*g/dL for her spouse. By October 2024, BLLs decreased to 8 *μ*g/dL for the spouse and to 3 *μ*g/dL for the child.

## Discussion

Substantial lead exposure was identified among members of a family associated with use of *kansa* and *pital* metalware for food and drinks. These types of traditional metalware products are widely used in South Asian communities for food and drink or in religious practices (*7*). Lead can be added to these types of metalware vessels to improve malleability or reduce production costs. Although *kansa* and *pital* are chemically distinct, the terms often are used interchangeably by community members. In Ayurveda, the ancient traditional medical system from India, *kansa* usage is believed to have health benefits (*7*).

These findings underscore evidence that traditional metalware can pose a considerable risk for lead exposure. A recent study tested traditional metalware imported from several countries and available for purchase in the United States (*8*). The metalware items were tested using simulated cooking and storage conditions, and the items, especially those from Afghanistan and India, leached lead at levels that exceeded recommended dietary limits. One of the metalware items with a high lead leachate level was a *pital* brass pot imported from India.

NYC DOHMH surveillance of local stores during 2017–2022 detected leachable lead at levels far exceeding reference limits in numerous samples of bronze and brass metalware from South Asia (*1*). U.S. federal regulations permit the sale of such products if they are correctly labeled stating that the article is not for food use (*4*). Although NYC DOHMH has taken enforcement actions to restrict businesses from selling similar products without correct labeling, this requirement might not be sufficient to prevent persons from using these culturally ingrained products for food and drinks. Also, the labels often are only in English, limiting accessibility to non-English speakers. Moreover, regulatory restrictions are limited to products being sold in the United States, which excludes items that are personally hand-carried into the United States from abroad, as was the case for the metalware items described in this report.

Although the affected family adhered to NYC DOHMH’s recommendation to stop using the metalware, risk communication can be challenging, especially when addressing risk factors with cultural significance. Among certain communities, these products are culturally integrated and passed down through generations. The items often are perceived as safe, or even beneficial for health and well-being. Raising awareness among communities about the lead risks associated with use of these products can help reduce exposure. NYC is an ethnically diverse city, and NYC DOHMH’s blood lead surveillance program provides an opportunity to identify lead-containing consumer products from around the world. Even small case investigations can reveal previously unrecognized lead sources and be important contributions to improving public health. Broader collaboration among public health agencies, clinical providers, and governmental and nongovernmental entities are needed to develop and implement strategies for reducing global lead exposures from traditional consumer products.

### Implications for Public Health Practice

Clinicians and public health professionals should be aware that certain imported metalware from around the world can be communal sources of lead exposure when used to prepare or serve food and drinks. Knowing that lead might be added to objects that have cultural significance, such as *kansa* and *pital* metalware, can aid in the identification of lead sources. These types of metalware products are frequently used within the South Asian community, which is already at risk for potential lead exposures from other traditional consumer products (*1*,*5*,*9*,*10*).

Health care providers, in partnership with public health officials, can serve as trusted sources of health information, by counseling families on how to reduce exposures to known lead sources. Health care providers also play an important role in testing BLLs of persons at risk for exposure to lead. The role of clinicians varies by jurisdiction. In NYC, where testing of young children is mandated, clinicians are required to conduct blood lead tests and report the findings; NYC DOHMH primarily oversees the identification of lead sources in homes and offers guidance for addressing them. When testing is based on a risk assessment, as it is in NYC for children aged >2 years and pregnant persons, or in jurisdictions where routine testing is not mandated, clinicians must be especially vigilant in identifying lead risk factors and testing those persons at risk for exposure. This report highlights the importance of considering testing other household members when one person is found to have an elevated BLL, especially when imported products might be potential communal sources of lead exposure.

## Footnotes

*
Lead - ToxFAQs - Agency for Toxic Substances and Disease Registry

†
Lead Exposure in Pregnancy - NYC DOHMH

§
Lead Exposure in Children - NYC DOHMH

¶
Occupational Safety and Health Standards for Lead - OSHA

**
Blood Lead Level Guidance - CDC

††
 Flame Atomic Absorption Spectrophotometry - EPA

§§
Inductively Coupled Plasma - Mass Spectrometry - EPA

¶¶
Standard Test Method for Lead and Cadmium Extracted from Glazed Ceramic - ASTM International
