# *Erratum:* Vol. 74, No. 18

**DOI:** 10.15585/mmwr.mm7425a2

**Published:** 2025-07-10

The report “Elevated Blood Lead Levels in a Pregnant Woman and her Family from Traditional *Kansa* (Bronze) and *Pital* (Brass) Metalware — New York City, 2024” contained several errors.

On page 298, the fourth sentence of the Abstract should have read, “Use of these metalware items for preparing and serving food and drinks was associated with blood lead levels above **CDC’s** blood lead reference value of 3.5 *µ*g/dL in a pregnant woman, her spouse, and their child (range = 6–18.7 *µ*g/dL). The fourth sentence of the Methods section should have read, “Follow-up actions **by NYC DOHMH** are initiated at threshold blood lead levels (BLLs) of 3.5 *μ*g/dL for children and pregnant women and 5 *μ*g/dL for nonpregnant adults.**” The third footnote should have read, “^§^ Lead Exposure in Children - NYC DOHMH; Lead Exposure in Adults - NYC DOHMH.” The fifth footnote should have read “** Blood Lead Level Guidance - CDC; CDC Updates Blood Lead Reference Value | Childhood Lead Poisoning Prevention - CDC.”

On page 301, the second sentence of the Summary should have read, “In July 2024, blood lead screening in New York City identified a pregnant woman and two family members with blood lead levels above **CDC’s blood lead** reference value of 3.5 *µ*g/dL.”

